# Death of Professor Daniell

**Published:** 1845-06

**Authors:** 


*Death of Professor Daniell.*—It is our painful duty to record the awfully sudden death of Professor Daniell, a philosopher whose name has become a household word to the electrician, and will always be remembered in connection with his admirable discovery of the Constant Battery, and his other contributions to electrical science. As professor at King's College, London, he was engaged in delivering a course of lectures on "Current Affinity;" or Electro-Chemistry. We had the good fortune of being present at several of these lectures, but were unwillingly absent on Thursday the 13th of March. On the afternoon of that day he lectured with his accustomed good spirits and in his usual cheerful manner, and concluded his lecture a few minutesbefore four o'clock, in order to be present at his place at the council-board of the Royal Society, of which he was foreign secretary. Before leaving the lecture room, he conversed with his known courtesy with some who were present; and making some lively remarks to a student, who was interested in the subject of the lecture, he went to the Royal Society's apartments, which are at the Somerset House, close at hand. Within less than an hour from this time he was no more. Dr. Bowman, who sat opposite to him at the council-table, observed a strange fixedness in his eyes, and at first thought him to be in a fit. With the concurrence of those present, he opened a vein in the neck, but it was of no avail; the hand of death was upon him, and all human means were powerless. A few minutes elapsed, and all was over. The cause of his death was apoplexy, to which he had a constitutional tendency. His age was fifty-five years.—*Electrical Magazine.*

